# Efficiency of Ozonated Water Treatment with a Microbubble System for Sanitization and Preservation of Postharvest Quality of Acerolas

**DOI:** 10.3390/foods14101814

**Published:** 2025-05-20

**Authors:** Carollayne Gonçalves-Magalhães, Lêda Rita D’Antonino Faroni, Paulo Roberto Cecon, Ernandes Rodrigues de Alencar, Marcus Vinícius de Assis Silva, Alessandra Aparecida Zinato Rodrigues, Handina da Graça Lurdes Langa Massango, Marcia Joaquim da Silva

**Affiliations:** 1 Department of Agricultural Engineering, Universidade Federal de Viçosa, Viçosa 36570-900, MG, Brazil; carollayne.magalhaes@ufv.br (C.G.-M.); ernandes.alencar@ufv.br (E.R.d.A.); marcus.assis@ufv.br (M.V.d.A.S.); alessandra.rodrigues@ufv.br (A.A.Z.R.); handina.langa@ufv.br (H.d.G.L.L.M.); marcia.j.silva@ufv.br (M.J.d.S.); 2 Department of Statistics, Universidade Federal de Viçosa, Viçosa 36570-900, MG, Brazil; cecon@ufv.br; 3 Department of Chemistry, Universidade Federal de Viçosa, Viçosa 36570-900, MG, Brazil

**Keywords:** *Malpighia emarginata* DC., ozone sanitization, fruit storage, microorganism inactivation, physicochemical analyses

## Abstract

This study aimed to investigate the effectiveness of ozonated water in the sanitation and postharvest quality of acerola fruits. The experiment comprised seven treatments: a control group with untreated fruits, three different durations of exposure to ozone microbubbles (20, 40, and 60 min), and three different durations of exposure to ozone-free microbubbles (20, 40, and 60 min). Acerola fruits were stored in a refrigerated environment below 5 °C at 87% relative humidity. Microbiological and quality analyses were performed immediately after ozonation on day 0 and then on storage days 3, 6, and 9. The quality parameters assessed included the fresh mass loss percentage, firmness, soluble solid content, pH, total titratable acidity, vitamin C, color, total phenolic compounds, and total antioxidant activity. The use of ozonated water was found to effectively maintain the firmness of the acerolas, regardless of the exposure duration. Changes were observed on the surface of fruits treated with ozone microbubbles, especially when 60 min of exposure was adopted. Treating acerolas with ozone microbubbles for 20 min proved to be the best condition for inactivating bacteria and fungi and preserving the vitamin C, pH, total titratable acidity, total phenolic compounds, and total antioxidant activity of the fruits throughout storage. In conclusion, ozonated water is a promising technology for sanitizing and preserving the postharvest quality of acerola.

## 1. Introduction

Considered a “superfruit” due to its high nutraceutical value, acerola (*Malpighia emarginata* Sessé & Moc. ex DC.) contains interesting bioactive compounds including antioxidants, phenols, flavonoids, anthocyanins, carotenoids, and minerals [1,2,3]. These compounds have antihyperglycemic and anticancer effects and contribute to protecting the skin barrier, promoting weight loss, boosting immunity, and preventing iron genotoxicity [4,5,6,7]. The vitamin C content in these fruits is remarkably high, ranging from 1000 to 4000 mg 100 g^−1^ of pulp, depending on the variety and ripeness, which is 20 to 30 times higher than the ascorbic acid content in citrus fruits such as oranges and lemons [8,9].

Brazil is the world’s largest producer, consumer, and exporter of acerolas. Nonetheless, the consumption of fresh acerolas is low due to the high perishability of the fruits [10,11]. The species has uneven flowering, making it difficult to determine the harvest point [12,13]. The estimated postharvest loss is around 40% of the production [14,15]. Since it is a climacteric-ripening fruit, it experiences significant moisture loss, leading to accelerated senescence and deterioration [10]. The fragile skin of the fruits provides little protection against physical damage during transport and external injuries when stored at temperatures below 5 °C or above 20 °C and a relative humidity under 85% [16] (pp. 27–47). These changes decrease the commercial value of the fruit and make it more susceptible to postharvest rotting, typically caused by the fungi *Alternaria* spp., *Fusarium* spp., *Aspergillus* spp., *Penicillium* spp., and *Colletotrichum gloeosporioides* [16] (pp. 27–47). The challenges in the harvest and postharvest restrict the distribution of acerolas to local areas near the cultivation sites or require processing to minimize losses, thereby limiting the marketing opportunities for fresh fruit. The production of acerola in Brazil was approximately 61 thousand tons in 2017 (latest data to date), generating a revenue of over BRL 90,000.00 [17,18]. According to these authors, more than 50% of the acerola produced in Brazil is destined for industrial processing, depending on the region of cultivation.

After harvesting, the fruit washing and sanitization steps are essential. These steps remove dirt and pesticide residues and reduce the microbial load on the fruits [19]. The food sanitization process can be physical or chemical [20]. Physical sanitization includes treatment using heat, gamma irradiation, UV light, and ultrasound. In chemical sanitization, the use of peracetic acid, chlorine, chlorine dioxide, hydrogen peroxide, and ozone stand out [21]. Currently, chlorine solutions are the most widely used sanitizers in the world due to their broad antimicrobial action, easy application, and low cost [22,23]. However, there is the possibility of the formation of carcinogenic chlorinated compounds in water, such as trihalomethanes (THMs) and haloacetic acids (HAAs), when chlorinated compounds react with organic matter [24]. In this scenario, the use of ozonated water has been studied as an alternative to the chlorinated compounds that have been researched, where the use of ozonated water stands out [25].

In recent years, ozone (O_3_) has stood out as an efficient and sustainable alternative for the postharvest treatment of fruits. It has one of the strongest oxidative potentials among the oxidizing agents, second only to fluoride [26]. When ozone decomposes, it turns into oxygen, exempting the treated products from harmful residues and the environment from toxic waste [27,28]. This behavior classifies ozone as a generally recognized as safe (GRAS) substance by the United States Department of Agriculture (USDA) [27,29], and these characteristics make ozone a superior alternative to conventional sanitizers like chlorine, which produce harmful by-products [30].

When applied by immersion in ozonated water, the ozone antimicrobial activity can be intensified if associated with microbubbles dimensioned from 1 to 100 µm (especially under 50 µm). The microbubbles facilitate ozone solubility and increase the retention time and the relationship between the surface and volume [31]. Microbubbles are typically injected into the water through a turbulent flow system, mechanical shear, pressurized dissolution, or microbubble generators with forced bubble detachment [32]. Previous studies have indicated the efficiency of ozonated water produced with a microbubble system in removing pesticides and preserving the quality of apples [33], strawberries, cherries, and apricots [34].

In this sense, using ozonated water in a microbubble system represents an efficient alternative for sanitizing acerolas. To date, there have been no scientific studies on the use of ozone for the disinfection and maintenance of the physicochemical quality of fresh acerolas throughout storage. Considering the attested efficiency of ozone in sanitizing and prolonging the shelf life of other highly perishable fruits and vegetables, this study aimed to determine the effects of ozonated water with a microbubble system on the sanitization and postharvest quality of fresh acerolas.

## 2. Materials and Methods

### 2.1. Obtaining the Fruits and Initial Characterization

Acerolas were obtained from a family farm in Viçosa, Minas Gerais, Brazil. Only one harvest was carried out to obtain the fruits used in the experiment. Ripe fruits were harvested at maturation stages 4 (60% pink-orange and 40% red skin) and 5 (uniform red skin) [16] (pp. 27–47) and were placed inside cardboard boxes, protected from sunlight, and promptly transferred to the postharvest laboratory. The acerolas used in the experiment did not receive any previous postharvest treatment. Before the ozonation process, the fruits were selected based on their external color, and those with damaged skins were discarded. They underwent an initial physicochemical characterization to assess the total soluble solid content and pulp color. Fruits from the same farm were used to ensure uniformity of the fruit in the batch, especially concerning the microbial load. It was not possible to use fruits from only one ripening stage, since the acerola is a species with uneven flowering. The fruits harvested were at ripening stages 4 and 5.

### 2.2. Ozone Treatment Settings

The experiment was conducted using a split-plot design, with the whole plot corresponding to the treatments (ozone microbubble, ozone-free microbubble, and control) and the subplots to the storage times (0, 3, 6, and 9 days). It followed a completely randomized design (CRD) with three repetitions. The assays comprised seven treatments: a control group (untreated fruits; T1), three exposure times to ozonated water and microbubble system at 5.60 mg L^−1^ of O_3_ (20, 40, and 60 min; T2, T3, and T4, respectively), and three exposure times to ozone-free microbubbles (20, 40, and 60 min; T5, T6, and T7, respectively). In the ozone-free microbubble treatments, only the oxygen concentrator and the microbubble generator remained turned on.

It is important to mention that the treatments adopted in the present study were defined in preliminary tests (Tables S1–S4 in the Supplementary Materials). In the preliminary tests, the fruits were exposed to ozone microbubbles, with an input gas concentration of similar, and ozone-free microbubbles for, 5, 10, 20, and 30 min, considering the effect on the counts of aerobic mesophiles and filamentous fungi and yeasts. The results of the preliminary tests demonstrated a greater efficiency of ozone microbubbles in controlling the aerobic mesophiles, at times of 20 and 30 min (Table S1). Thus, 20 min was adopted as the minimum period for the treatment of the fruits. Exposure periods of 40 and 60 min were also adopted to determine a possible increase in the effectiveness of inactivating microorganisms and the occurrence of undesirable effects on fruit quality.

Ozone gas was obtained using a concentrator model M10^®^ (myOzone, Jaguariúna, São Paulo, Brazil), which had a production capacity of 10 g h^−1^ at 25 °C via the corona effect (dielectric barrier discharge, DBD). Ozone gas was produced using oxygen (90% purity) from an oxygen concentrator, model EverFlo™ OPI 5LPM, number 15668 (Philips Respironics, Murrysville, PA, USA). The volumetric oxygen flow supplied to the ozone generator was 1.5 L min^−1^, measured with a flowmeter model MF5700^®^ (Siargo Ltd., Chengdu, China). The ozone input and output concentrations were assessed via the indirect titration iodometric method [35], as specified by the International Ozone Association (IOA).

**Figure 1:**
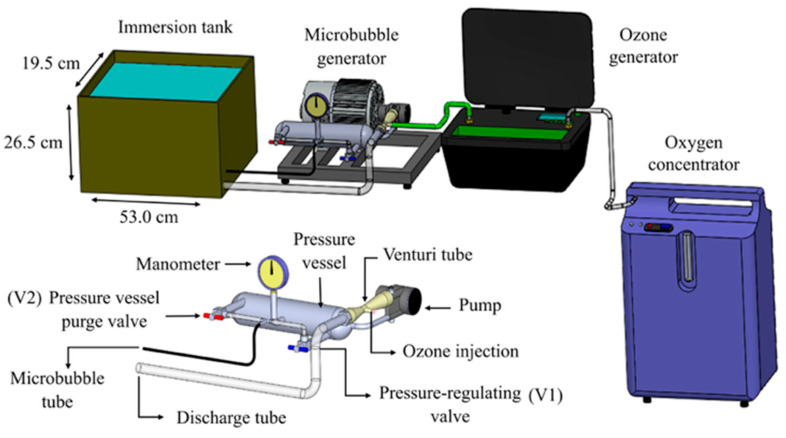
Experimental setup for water ozonation incorporating ozone with a microbubble generator.

Ozone or oxygen was incorporated into the water using a microbubble generator, model MB600^®^ (myOzone, Jaguariúna, São Paulo, Brazil) (Figure 1). The device consisted of a pump connected to a pressure vessel. At the pump inlet, a Venturi tube-type injector introduced ozone or oxygen into the system. The ozone or oxygen gas was directed into the pressure vessel, whose inner pressure was regulated by a valve (V1) that restricted the water flow until the system reached the working pressure of 8.43 kgf cm^−2^. At this condition, the circulating volumetric flow rate of water was 0.12 m^3^ h^−1^. Inside the pressure vessel, the gas bubbles that formed during the injection by the Venturi tube were fractionated into microbubbles. In order to prevent gas buildup, a valve (V2) released excess ozone microbubbles or ozone-free microbubbles that had not been incorporated into the water. The pump discharge was directed into the tank where the acerola fruits were submerged. For each acerola treatment, 15 L of water was used. This volume was held in a 30-L tank (53.0 cm length × 19.5 cm width × 26.5 cm height) connected to the microbubble generator (Figure 1). The ozone concentration at the generator output was 12 mg L^−1^, resulting in 5.68 ± 0.08 mg L^−1^ of the gas dissolved in water after 15 min (a 46.66% incorporation).

During the ozone microbubble or ozone-free microbubble treatment, the water temperature was monitored and maintained at approximately 7 ± 1 °C. For each treatment, 1.1 kg of acerolas were packed in plastic nets and immersed in water. The first net was taken out of the tank after 20 min, the second after 40 min, and the third after 60 min. After the treatment with ozone microbubbles or ozone-free microbubbles, the fruits were laid onto a previously sanitized table for drying at room temperature (25 °C) for 30 min.

### 2.3. Acerola Storage After Ozonation

The acerola samples subjected to ozone microbubbles, ozone-free microbubbles, or the control treatment were weighed and identified according to the treatment. Each experiment plot consisted of one tray containing 80 ± 0.5 g of acerola. The fruits were stored for nine days in a refrigerated environment at 5 °C and 87% relative humidity. Microbiological and physicochemical analyses were conducted immediately post-treatment (day 0) and after storage days 3, 6, and 9.

### 2.4. Microbiological Analyses

Microbiological analyses were carried out following the criteria of the International Organization for Standardization (ISO) 6887-1/1999 [36] and in accordance with the American Public Health Association (APHA) [37]. A total of 10 g of the samples was used from each replication of the different treatments. Each sample (10 g) was transferred to sterilized plastic bags, macerated, and diluted in 90 mL of a previously sterilized saline solution at 2%, resulting in a 10^−1^ dilution. From this first dilution, it was possible to obtain the subsequent ones. The aerobic mesophilic count considered the dilutions from 10^−2^ to 10^−4^, while the filamentous fungi and yeast count used the dilutions from 10^−2^ to 10^−5^. Microbiological analyses were performed in a vertical laminar-flow hood, model PCR FLV-1266/4 (Filterflux, Piracicaba, São Paulo, Brazil).

The mesophiles were inoculated using the pour-plate method. Aliquots of 1 mL were pipetted into a Petri dish, and then a liquid culture medium (plate count agar—PCA) was added. The spread-plate method was used to inoculate the filamentous fungi and yeasts. Aliquots of 0.1 mL (100 µL) were pipetted into a solid culture medium (potato dextrose agar—PDA) acidified with 10% tartaric acid. Subsequently, the material was spread using a Drigalski loop.

The plates were incubated inside biochemical oxygen demand (BOD) chambers. Those containing mesophiles were kept inside the BOD chamber for 48 h (2 days) at 30 °C, while those with filamentous fungi and yeasts were incubated for 120 h (5 days) at 25 °C. After the incubation period, the microorganisms were counted, considering the ranges of 25–250 colonies for aerobic mesophiles and 15–150 colonies for filamentous fungi and yeasts. The results were expressed in log CFU g^−1^.

### 2.5. Physicochemical Quality Analysis

The physicochemical evaluations adhered to the criteria recommended by the Adolfo Lutz Institute [38] and the International Commission on Illumination [39]. Analyses of the total phenolic compounds and total antioxidant activity were performed according to the methodology of Asami et al. [40] with modifications. The seeds, peduncle, and floral receptacle were removed before measurements were taken for the soluble solid content, pH, total titratable acidity, vitamin C, pulp color, total phenolic compounds, and total antioxidant activity. The rest of the material was crushed using a hand blender, model RI1341 (Philips Walita, Varginha, Minas Gerais, Brazil). Firmness, pH, total titratable acidity, vitamin C, total phenolic compounds, and total antioxidant activity were analyzed in triplicate, and soluble solid content and pulp color in quadruplicate.

#### 2.5.1. Fresh Mass Loss (%)

The fresh mass loss percentage by each evaluation day was calculated by subtracting the initial mass of each sample before the experiment (day 0) from the mass after 3, 6, and 9 days of storage. The result was multiplied by 100 and divided by the initial mass (Equation (1)). The samples were weighed with a semi-analytical digital scale, model BK 8000 (Gehaka, São Paulo, Brazil), which had an accuracy of 1 × 10^−2^.

(1) $$\mathrm{WLt}\left(\%\right)=\frac{W_{0}\;-\;\mathrm{Wt}}{W_{0}}\;\times \;100$$

where WLt (%) represents the percentage of fresh mass loss by each evaluation day, W_0_ corresponds to the initial sample mass on day 0, and Wt is the sample mass on the evaluation day.

#### 2.5.2. Firmness (Newton)

Three acerola fruits were randomly selected from each plot and measured for firmness using a digital fruit hardness tester, model PTR 300 (Instrutherm, São Paulo, Brazil). A 3-mm penetrometer tip was used to pierce the fruits at four points: longitudinally (at the base and apex) and transversely (from each side). The compression force was expressed in Newton (N).

#### 2.5.3. Total Soluble Solid Content

The soluble solid content was quantified with a digital refractometer, model Pocket PAL-3 (Atago, Tokyo, Japan), with four readings taken per plot. A drop of crushed pulp was used for the readings, and the final values were expressed in %.

#### 2.5.4. Potential of Hydrogen (pH)

A portable digital pH-meter, model K39-0014PA (Kasvi, São José dos Pinhais, Brazil), was used to determine the pH. The readings were taken in a solution containing 5 g of crushed pulp and 50 mL of distilled water.

#### 2.5.5. Total Titratable Acidity (Citric Acid)

The potentiometry method was employed to measure the total titratable acidity of the acerolas (percentage of citric acid per 100 g of pulp, citric acid % 100 g^−1^). The analyses used a portable digital pH-meter, model K39-0014PA (Kasvi, São José dos Pinhais, Brazil). A mixture of 5 g of crushed pulp and 50 mL of distilled water was titrated with a standard sodium hydroxide solution (NaOH 0.1 mol L^−1^) until reaching pH 8.2. The total titratable acidity was calculated by multiplying the volume of sodium hydroxide used for titration by its correction factor and by 10. The result was then divided by the sample mass (Equation (2)).

(2) $$\mathrm{Citric}\;\mathrm{acid}\;(\%\;100\;g^{-1}\;\mathrm{of}\;\mathrm{pulp})=\frac{V\;\times \;f\;\times \;10}{m}\;$$

where V represents the volume (mL) of sodium hydroxide (NaOH 0.1 mol L^−1^) used in the titration, f is the correction factor, and m is the sample mass (g).

#### 2.5.6. Vitamin C (Ascorbic Acid)

Vitamin C was obtained by titration using a potassium iodate solution (KIO_3_) with a stoichiometric factor of 8.806. The results were expressed in grams of ascorbic acid per kilogram of pulp (milligram of ascorbic acid per 100 g of pulp, ascorbic acid mg 100 g^−1^). The iodate was titrated in a solution containing 10 g of crushed pulp, 50 mL of distilled water, 10 mL of sulfuric acid 20%, 1 mL of potassium iodide 10%, and 1 mL of starch 1%. The endpoint was reached when the indicator (starch) changed to brown-violet. The volume (mL) of potassium iodate spent on titration was used to calculate the levels of ascorbic acid (Equation (3)).

(3) $$\mathrm{Ascorbic}\;\mathrm{acid}\;(\mathrm{mg}\;100\;g^{-1}\;\mathrm{of}\;\mathrm{pulp})=\frac{100\;\times \;V\;\times \;f}{m}\;$$

where V corresponds to the volume (mL) of potassium iodate used in the titration, f is the stoichiometric factor of the potassium iodate solution, and m is the mass of the sample (g).

#### 2.5.7. Pulp Color

Crushed pulp from each plot was poured into transparent plastic containers, and four readings were taken, two on each side. The color readings were performed with a digital colorimeter, model CR-400 (Konica Minolta, Tokyo, Japan). Obtaining the coordinates L*, a*, and b* enabled the determination of color difference-Dif* (∆E*) (Equation (4)), hue (h*) (Equation (5)), and chroma (C*) (Equation (6)).

(4) $${∆E}^{*}=\sqrt{{{\left(L^{*}-{\;L}_{0}^{*}\right)}^{2}+(a^{*}-\;a_{0}^{*})}^{2}+{\left(b^{*}-\;b_{0}^{*}\right)}^{2}}$$

(5) $$h*=\arctan(\frac{b*}{a*})$$

(6) $$C*=\sqrt{\left({a*}^{2}+{b*}^{2}\right)}$$

The coordinate L* represents the white-black lightness, a* corresponds to the red-green color intensity, and b* is the yellow-blue color intensity. The coordinates L*, a*, and b* obtained in the initial characterization of the pulp are represented by L_0_, a_0_, and b_0_. The color difference on day 0 (∆E*) was calculated in relation to the average values of L*, a*, and b* from the initial characterization.

#### 2.5.8. Total Phenolic Compounds and Total Antioxidant Activity

##### Acerola Extract Preparation

Acerola extracts were prepared by mixing 1 g of processed pulp with 20 mL of the extractive solution, composed of distilled water, methanol, and acetic acid in the proportion 30:70:5 *v*/*v*/*v*. The extracts were homogenized for 20 min in a shaking incubator, model SL222 (Solab, Piracicaba, São Paulo, Brazil), and then centrifuged for 20 min at 560× *g* in a centrifuge, model Q222TM2 (Quimis, Diadema, São Paulo, Brazil). The resulting supernatant was used to prepare the dilutions. The total phenolic compounds and total antioxidant activity were analyzed by colorimetry and spectrophotometry using a spectrophotometer, model Cirrus 80 ST (Femto, São Paulo capital, São Paulo, Brazil).

##### Total Phenolic Compounds (Gallic Acid)

The total phenolic compounds were measured using the Folin–Ciocalteu colorimetric method, which involves the oxidation of phenolates into a blue Mo-W complex. The Folin–Ciocalteu solution was diluted in distilled water at a ratio of 1:10 *v*/*v*. The acerola extracts were diluted tenfold in distilled water (1 mL of the extractive solution to 9 mL of distilled water). Then, 0.6 mL of the diluted solution was transferred to a Falcon tube, and 3 mL of the Folin–Ciocalteu solution was added. The mixture was stirred and left to stand for 3 min, then 2.4 mL of saturated sodium carbonate solution (7.5% *w*/*v*) was added. The tubes were stirred again and left to stand for 2 h in the dark. The analytical curve was plotted using a stock solution of gallic acid at 200 mg L^−1^. The wavelength of 760 nm was used in the spectrophotometric readings. The content of total phenolic compounds was determined by interpolating the absorbance of the samples against a calibration curve created using a gallic acid standard (0, 30, 60, 90, 120, and 150 µM). The findings were reported in grams of gallic acid per kilogram of pulp (milligram of gallic acid per 100 g of pulp, mg 100 g^−1^ of pulp). Equation (7) was used to calculate the total phenolic compounds.

(7) $$\mathrm{Gallic}\;\mathrm{acid}\;(\mathrm{mg}\;100\;g^{-1}\;\mathrm{of}\;\mathrm{pulp})=\frac{\mathrm{GAE}\;x\;100}{D.\mathrm{exrtract}}$$

where GAE stands for the gallic acid equivalent (obtained via the analytical curve), and D.extract corresponds to the dilution of the sample (g) or determination trial (g).

##### Total Antioxidant Activity According to the ABTS and DPPH Methods

The antioxidant activity was determined considering the ability to scavenge the free radicals ABTS (2,2′-azino-bis/3-ethylbenzothiazoline-6-sulfonic acid) and DPPH (2,2-diphenyl-1-picrylhydrazyl). Following the ABTS method, the acerola extract was diluted twice in distilled water using a 10 mL volumetric flask (5 mL extractive solution to 5 mL distilled water). Next, a 30-microliter aliquot was transferred to a Falcon tube, and 3 mL of the ABTS solution was added. For the DPPH method, the acerola extract was diluted tenfold in distilled water (1 mL of the extractive solution to 9 mL of distilled water). After dilution, an aliquot of 150 µL was mixed with 5850 mL of the DPPH solution in a Falcon tube.

Before taking the spectrophotometric readings of ABTS and DPPH, the mixtures were homogenized to stabilize the reaction. After that, they were incubated in the dark for 6 and 15 min, and the readings were taken at the wavelengths of 734 nm and 515 nm (ABTS and DPPH, respectively). In both methods, analytical-grade ethanol was used as the blank. The antioxidant activity was assessed using a calibration curve created with a standard solution of Trolox (6-hydroxy-2,5,7,8-tetramethylchroman-2-carboxylic acid). The Trolox standard was used at 100, 500, 1000, 1500, and 2000 µM (ABTS) and 50, 100, 200, 400, 600, 800, and 1000 µM (DPPH). Equation (8) calculated the antioxidant activity using the ABTS and DPPH methods, and the results were expressed in micromoles of Trolox per kilogram of pulp (micromole of Trolox per 100 g of pulp, µM 100 g^−1^ of pulp).

(8) $$\mathrm{ABTS}\;\mathrm{and}\;\mathrm{DPPH}\;\left(\mathrm{Trolox}\right)=\frac{\left[X\right]\mathrm{Trolox}\;\left(µM\right)\;x\;\mathrm{Total}\;\mathrm{extract}\;\mathrm{volume}\;(\mathrm{mL})}{\mathrm{Sample}\;\mathrm{mass}\;(g)}\;$$

### 2.6. Statistical Analysis

The data were evaluated via analysis of variance (ANOVA) and regression analysis. The means were compared by applying Tukey’s test at a 5% probability level using the Sisvar 5.6 software. The control group was compared with the other treatments using Dunnett’s test at a 5% significance level using the software Assistat 7.7. The regression models were chosen based on the significance of the regression coefficients according to the Student’s *t*-test. The test was applied at either a 1% or 5% probability level to the coefficient of determination (R^2^/r^2^ = $\frac{\mathrm{SQReg}}{\mathrm{SQTrat}}$) and to assess the phenomenon’s behavior. This analysis was handled by the Saeg 9.1 software. Regardless of the significance of the highest-order interaction, we decided to unfold it due to the interest of the research.

## 3. Results

### 3.1. Disinfection of Acerolas

At time zero, the aerobic mesophile count in the ozone microbubble treatments (T2 = 20 min, T3 = 40 min, and T4 = 60 min) and ozone-free microbubble treatments (T5 = 20 min and T6 = 40 min) were below 4.00 log (CFU g^−1^), with differences of 0.77, 0.72, and 0.99 log CFU g^−1^ for the ozone microbubbles (T2, T3, and T4, respectively) and 0.66 and 0.71 log CFU g^−1^ for the ozone-free microbubbles (T5 and T6, respectively) compared with the control (T1) (Table 1). By the third storage day, the 60-min treatment with ozone-free microbubble (T7) showed the highest bacterial incidence, registering 5.09 and 5.10 log (CFU g^−1^) on days 0 and 3, respectively. Among the ozone treatments (T2, T3, and T4), the lowest bacterial contamination throughout storage (days 3, 6, and 9) corresponded to the application for 20 min (T2). Effects of ozone on the count of these microorganisms were also observed during storage, especially when the exposure period of 20 min (T2) was adopted. For this treatment, values lower than 2.0 log CFU g^−1^ were observed in the count for aerobic mesophiles, after three days of storage. At the end of storage (day 9), a lower count of aerobic mesophiles was also observed in the T2 treatment, with differences of 1.44 and 0.83 log CFU g^−1^ in relation to the ozone microbubbles for 40 and 60 min (T3 and T4), respectively.

The lowest incidence of total molds and yeasts during storage was verified in the ozone treatment for 60 min (T4), which showed values of 4.45, 4.63, and 4.73 log (CFU g^−1^) on days 3, 6, and 9 (Table 1). However, the 20-min ozone microbubble treatment (T2) was found to be the most effective, as molds and yeasts were only detected from day 6 of storage. In the 60-min (T4) and 40-min (T3) treatments, these microorganisms were observed from days 0 and 3, respectively.

The results indicated that all ozone microbubble treatments (T2, T3, and T4) were more effective in reducing the growth of bacteria and fungi throughout storage compared with the ozone-free microbubble treatments (T5, T6, and T7) and the control (T1) (Table 1). This is because, from day 6 of storage, it was no longer possible to quantify the microorganisms in those treatments (ozone-free microbubble and control) by the chosen technique, as the maximum limit of colonies had been exceeded.

The results indicate that longer ozone exposure (T2, T3, and T4) resulted in earlier fungal incidence during storage (Table 1). On day 0, the 60-min exposure to either ozone microbubbles (T4) or ozone-free microbubbles (T7) presented fungal contamination, with values of 4.32 and 4.41 log (CFU g^−^^1^), respectively. In the other treatments (T1, T2, T3, T5, and T6), filamentous fungi and yeasts were observed on day 0 below the counting range (15–150 colonies), with values lower than 2.00 log (CFU g^−^^1^). In the treatments with ozone microbubbles (T2, T3, and T4), microbiological contamination was only countable at the exposure times of 40 and 20 min (T3 and T2), starting from days 3 and 6 of storage, respectively.

### 3.2. Physicochemical Quality

#### 3.2.1. Fresh Mass Loss, Firmness, Total Soluble Solids, Potential of Hydrogen, Total Titratable Acidity, and Vitamin C

Table 2, shows the average values for fresh mass loss (%), firmness (N), total soluble solids (%), potential of hydrogen (pH), total titratable acidity (citric acid % 100 g^−^^1^), and vitamin C (ascorbic acid mg 100 g^−^^1^) of the acerolas throughout storage. The treatments had significant effects (*p* < 0.05) on these variables, except for fresh mass loss. The variables pH, total titratable acidity, and vitamin C displayed variations among the treatments (T1 to T7) and in relation to the control group (T1) on all assessment days.

In all treatments (T1 to T7), the fresh mass loss was less than 2.0% by day 3 of storage, approximately 2.5% by day 6, and around 4.0% by day 9 (Table 2). Significant effects (*p* < 0.05) were verified on the firmness of the fruits only by days 3, 6, and 9 of storage. The ozone microbubble treatment for 60 min (T4) exhibited the highest firmness averages throughout storage. When compared with the control (T1), differences were observed in the ozone-free microbubble treatments (T5, T6, and T7) after storage day 3 and in the ozone treatments (T2, T3, and T4) after days 6 and 9. Immediately after ozonation (day 0), all treatments (T1 to T7) showed firmness values close to 0.50 N. From storage day 3, the firmness was significantly decreased, especially between days 6 and 9. At the end of the storage period (day 9), the compression force ranged from 0.25 to 0.37 N in the ozone microbubble treatments (T2, T3, and T4), and it was equal to or below 0.14 N in the other treatments (T1, T5, T6, and T7).

During the evaluation period, the total soluble solid content was close to 9.00 or 10.00% in all treatments (T1 to T7) (Table 2). Peaks were observed on storage day 3 when the total soluble solid content ranged from 10.30 to 11.27%. The highest averages were obtained in the 20-min ozone-free microbubble treatment (T5) between days 3 and 9. The treatments differed from the control group (T1) at time 0 and on days 6 and 9 of storage. On the third day of storage, the pH of the acerolas was below 3.00 in all treatments (T1 to T7) (Table 2). Between the sixth and ninth days of storage, the pH values increased up to 3.10. As the pH rose throughout storage, the total titratable acidity generally decreased. At the beginning (day 0), the total titratable acidity was above 2.00 citric acid % 100 g^−^^1^ in all treatments, and by day 9, it was equal to or below 1.87 citric acid % 100 g^−^^1^.

Immediately after ozonation (day 0), all treatments (T1 to T7) displayed a vitamin C content above 1300.00 ascorbic acid mg 100 g^−^^1^ (Table 2). On that evaluation day, the lowest averages corresponded to the ozone-free microbubble treatments (T5, T6, and T7), while the highest ones were found in the control group (T1) and ozonated water treatments (T2, T3, and T4). When analyzing the effects of ozone microbubbles (T2, T3, and T4) and ozone-free microbubbles (T5, T6, and T7), it was observed that between days 3 and 9 of storage, the vitamin C levels were the highest in the 20-min ozone microbubble treatment (T2). The ozone microbubble treatments for 20 and 40 min (T3 and T4) were the most efficient in preserving vitamin C within the optimal range, with the average values above 1000.00 ascorbic acid mg 100 g^−^^1^ on storage days 9 (T3) and 6 (T2). Using ozone-free microbubbles for 20, 40, and 60 min (T5, T6, and T7) or ozone microbubbles for 60 min (T4) preserved this variable until the third day of storage. These treatments were less effective than the control (T1), which maintained adequate levels of vitamin C for up to day 6 of storage.

The regression equations and their respective coefficients of determination (R^2^/r^2^) (Table 3) represent the variation of fresh mass loss (%), firmness, total soluble solids, pH, total titratable acidity, and vitamin C in the treatments (control, ozone microbubble, and ozone-free microbubble) as a function of storage and exposure times. The average was used to describe the treatments whose variables were not significantly affected by the storage and exposure times.

Fresh mass loss was only significantly influenced by the storage time (*p* < 0.01), showing a linear effect in both the ozone microbubble (T2, T3, and T4) and ozone-free microbubble (T5, T6, and T7) treatments (Table 3). On each evaluation day, the average increase in the percentage of mass loss in all T1 to T7 conditions was approximately 0.45%. Firmness exhibited quadratic effects in the ozone treatments (T2, T3, and T4) for both the storage (*p* < 0.01) and exposure times (*p* < 0.05). In the ozone-free microbubble treatments (T5, T6, and T7), this variable was significantly influenced solely by the storage time, presenting a linear effect for this factor (*p* < 0.01). The average firmness reduction verified in the ozone microbubble treatments (T2, T3, and T4) on each evaluation day was 0.014 N. This was lower than the firmness reductions in the ozone-free microbubble treatments (T5, T6, and T7) and the control (T1), which averaged losses of 0.036 N and 0.047 N, respectively.

The pH and total titratable acidity showed significant effects on the treatments with ozone microbubbles (T2, T3, and T4) and ozone-free microbubbles (T5, T6, and T7) only in relation to storage time (*p* < 0.01), displaying a linear behavior (Table 3). As for vitamin C, both the storage (*p* < 0.01) and exposure times (*p* < 0.01) significantly influenced this variable, with a linear effect in both cases. The least reduction in the ascorbic acid content was observed in the ozone microbubble treatments (T2, T3, and T4). The average decrease in vitamin C was 62.74 ascorbic acid mg 100 g^−^^1^ between consecutive evaluation days. This was lower than verified in the ozone-free microbubble (T5, T6, and T7) and control (T1) treatments, which decreased by 74.29 and 87.17 ascorbic acid mg 100 g^−^^1^, respectively. The coefficients of determination (r^2^) for vitamin C ranged from 0.8178 to 0.9398.

#### 3.2.2. Pulp Color, Total Phenolic Compounds, and Total Antioxidant Activity (ABTS and DPPH)

Table 4, contains the average values of color difference (Dif*), color hue (h*), color saturation (C*), total phenolic compounds (gallic acid mg 100 g^−1^), and total antioxidant activity ABTS and DPPH (Trolox µM 100 g^−1^) in the acerola pulp over storage. The variables were significantly influenced (*p* < 0.05) by the treatments. All characteristics exhibited differences throughout storage when comparing the ozone and ozone-free microbubble treatments (T2 to T7) with the control (T1).

Immediately after the treatments (day 0), the highest averages for acerola pulp color difference (Dif*) were observed in the ozone microbubble treatments (T2, T3, and T4) (Table 4). Between storage days 3 and 9, the 60-min ozone-free microbubble treatment (T7) resulted in higher averages of color difference (4.07, 6.60, and 14.23 Dif*) compared with the other treatments (T2, T3, T4, T5, and T6) and the control (T1). Regarding color hue, between storage days 3 and 6, all ozone (T2, T3, and T4) and ozone-free microbubble (T5, T6, and T7) treatments differed from the control (T1) by exhibiting higher average values of hue angle. By the end of the storage period (day 9), all ozone treatments (T2, T3, and T4) presented higher averages of color hue (40.44, 42.36, and 40.25 h*) compared with the ozone-free microbubble treatments (T5, T6, and T7) and the control (T1). As for color saturation, the fruits exposed to ozone (T2, T3, and T4) got more saturated after ozonation on day 0 (15.09, 13.80, and 14.83 C*). The 60-min ozone microbubble treatment (T4) showed the highest color saturation during storage, with average values of 17.09, 15.93, and 23.17 C* on evaluation days 3, 6, and 9, respectively. These values were higher than the other treatments (T1, T2, T3, T5, T6, and T7).

Immediately after the treatment (day 0), ozone microbubble exposure for 20, 40, and 60 min (T2, T3, and T4) exhibited higher bioavailability of total phenolic compounds compared with the control (T1) and ozone-free microbubble treatments (T5, T6, and T7), with values exceeding 2100.00 gallic acid mg 100 g^−1^ (Table 4). Also, on day 0, the highest antioxidant activity measured using the ABTS method was found in the ozone treatments (T2, T3, and T4) and ozone-free microbubble treatment for 60 min (T7) (9204.84 and 9323.78 Trolox µM 100 g^−1^). In contrast, the DPPH method revealed the highest antioxidant activity on day 0 when ozone was applied for 20 min (T2) (893.39 Trolox µM 100 g^−1^). As assessed by both methods from days 3 to 9 of storage, the ozone treatment for 20 min (T2) had higher average values of total phenolic compounds content and total antioxidant activity than the other ozone microbubble and ozone-free microbubble treatments (T3 to T7).

Table 5 presents the regression equations and coefficients of determination (R^2^/r^2^) for the color variables (difference/Dif*, hue/h*, and saturation/C*), total phenolic compounds (gallic acid mg 100 g^−1^), and total antioxidant activity via ABTS and DPPH methods (Trolox µM 100 g^−1^) as a function of storage and exposure times of acerola pulp subjected to the different treatments: control (T1), ozone microbubble (T2, T3, and T4), and ozone-free microbubble (T5, T6, and T7). The average was used to describe the treatments whose variables were not significantly affected by storage or exposure times.

Significant effects of the ozone treatments (T2, T3, and T4) on the three color variables and total phenolic compounds were found only for storage time (*p* < 0.01) (Table 5). The exposure times to ozone-free microbubble (T5, T6, and T7) significantly influenced solely the color difference and did not significantly affect the other variables. In this case, storage time had a quadratic effect (*p* < 0.01), while exposure time had a linear impact (*p* < 0.05). The total antioxidant activity assessed by both methods was significantly influenced by the exposure time to ozone (T2, T3, and T4). According to the ABTS method, there was a linear effect associated with storage time (*p* < 0.01) and a quadratic effect related to exposure time (*p* < 0.05). As for the DPPH method, a linear effect was observed in both storage (*p* < 0.01) and exposure times (*p* < 0.05).

On each evaluation day, the average reductions in the total phenolic compounds content were 331.36 and 106.28 gallic acid mg 100 g^−1^ in the ozone treatments (T2, T3, and T4) and the control (T1), respectively (Table 5). The antioxidant activity measured by the ABTS method showed average reductions per evaluation day of 314.96, 283.69, and 296.66 Trolox µM 100 g^−1^ in the treatments with ozone (T2, T3, and T4), ozone-free microbubble (T5, T6, and T7), and control (T1), respectively. In turn, the DPPH method exhibited average reductions per evaluation day of 59.71, 62.44, and 26.81 Trolox µM 100 g^−1^ in the ozone (T2, T3, and T4), ozone-free microbubble (T5, T6, and T7), and control (T1) treatments, respectively.

#### 3.2.3. External Appearance of Acerolas During Storage

**Figure 2:**
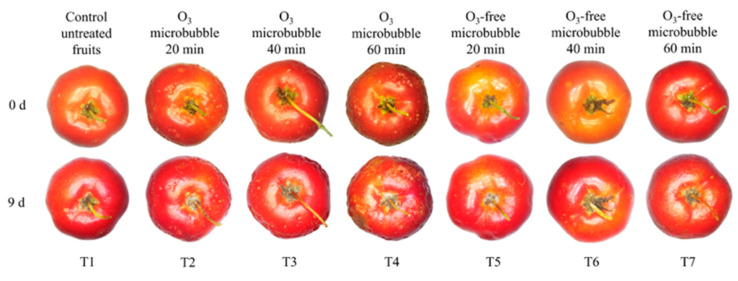
External appearance of the acerolas after 0 and 9 days of storage. Scale: 0.63× magnification using a microscope, model Leica S6D (Leica Microsystems Aotec, São Paulo, São Paulo, Brazil).

External changes were observed in acerolas immersed in ozonated water for all exposure periods (T2, T3, and T4). Remarkably, the most significant changes were verified after the 60-min ozone microbubble treatment (T4) (Figure 2). In general, the longer the exposure to ozone, the more perceptible the damage to the fruit skin. On the other hand, the exterior of the fruits submitted to the ozone-free microbubble treatments (T5, T6, and T7) and the control group (T1) showed no visible damage. However, sanitizing acerolas with ozone microbubbles (T2, T3, and T4) ultimately proved more efficient than the ozone-free microbubble treatments (T5, T6, and T7) and the control (T1) in reducing microbial contamination and preserving the fruits’ physicochemical characteristics.

## 4. Discussion

### 4.1. Disinfection of Acerolas

The role of ozone in controlling fungi and bacteria is associated with the progressive oxidation of phospholipids and proteins in the cell membrane [41]. Microorganisms are inactivated by the free radicals hydroxyl (OH-), hydroperoxyl (HO_2_), and superoxide (O_2_-), which are generated when ozone is applied in an aqueous medium [42]. Free radicals first react with the double bonds of lipids in the cell wall. Once this barrier is crossed, they react with outer lipopolysaccharides and inner lipoproteins, leading to increased membrane permeability [41]. As a result, the cell envelope ruptures, dispersing the cytoplasmic contents and genetic material (DNA and RNA), ultimately causing the death of the pathogen [43].

Some cases have reported a successful reduction in the bacterial and fungal contamination in fruits treated with ozone gas or ozone dissolved in water, but there have been no studies specifically targeting the use of ozone for fresh acerola sanitization. Strawberries treated with ozone gas at 14 mg L^−1^ for 5 min reduced the presence of the pathogenic bacteria *Salmonella enterica* and *Enterococcus faecium* on the fruit surface [29]. The efficiency of ozone gas applied at 9.9 to 29.7 mg L^−1^ for 10 to 60 min was also confirmed in controlling *Pseudomonas* bacteria in kiwis [44]. In turn, ozonated water at 0.8 mg L^−1^ for 40, 80, 120, and 160 min proved effective in reducing the incidence of *Colletotrichum gloeosporioides*, the fungus that causes anthracnose in papayas [45]. Ozonated water treatments were also successful in inactivating *Botrytis cinerea*, the causative agent of gray mold, in blueberries (10 and 18 mg L^−1^ for 10 to 30 min) [46] and strawberries (3.5 mg L^−1^ for 5 to 15 min) [47,48].

In the control of quality-indicator microorganisms, researchers found that immersing strawberries in ozonated water for 3 min [49,50] or exposing them to ozone gas (14 and 18 mg L^−1^ for 30 min) [51] reduced the presence of aerobic mesophiles, filamentous fungi, and yeasts during storage. In bananas treated with ozone gas (30 min) or ozonated water (0.36 mg L^−1^ for 10 min), the latter was found to be more efficient in lowering the total mold and yeast count and preserving the visual quality of the fruits for longer [52].

The trend observed during storage for filamentous fungi and yeasts may be linked to fungal quiescence. Some fungi can remain pathologically quiescent depending on the ripening phase of the fruit, only manifesting as climacteric respiration intensifies [53]. This behavior may also be related to the oxidation of the fruit epidermis due to prolonged contact with ozonated water. High concentrations and prolonged exposure can cause oxidation and discoloration of the fruit surface, making it more susceptible to external damage and creating a favorable environment for microorganism growth. These factors alter the composition of the fruits and speed up their senescence process [52,54].

The effects of ozone treatment (T2) on the aerobic mesophilic count in fruits, observed on the third and ninth days of storage (Table 1), may be associated with the damage caused by the gas to microorganisms. One hypothesis is that this damage does not result in the complete inactivation of microorganisms, but affects microbial growth during storage. Thus, the effect of ozone was observed during storage in treatment T2. As for treatments T3 and T4, which presented higher aerobic mesophilic counts, more pronounced damage to the surface of the fruits may have favored microbial growth, as damaged fruits are more susceptible to microorganisms [55].

A similar trend was observed in onions exposed to ozone gas (1.4 mg L^−1^ of ozone for 1, 3, and 5 min) [56] and strawberries treated with ozonated mist (20 and 40 mg L^−1^ of ozone input concentration for 5 and 10 min) [57]. In these studies, ozone also showed effects throughout storage on the inactivation of bacteria, indicating its latent effect with the ability to reduce bacterial growth during storage. Fungal quiescence may have been prolonged in some treatments due to the temperature and relative humidity conditions adopted in this research for acerola storage. These settings likely minimized respiration and moisture loss, explaining the behavior observed. In this case, it can be inferred that exposure to ozone gas for 60 min and exposure to ozone in an aqueous medium for 40 and 60 min may have reduced fungal quiescence, anticipating their proliferation in fresh acerolas.

### 4.2. Physicochemical Quality

#### 4.2.1. Fresh Mass Loss, Firmness, Total Soluble Solids, Potential of Hydrogen, Total Titratable Acidity, and Vitamin C

When marketing fresh fruits, preserving their fresh mass is paramount. In this study, the treatments did not affect the percentage of fresh mass loss in the acerolas, which may be explained by the temperature and relative humidity conditions in the storage environment. Adequate temperature and relative humidity settings reduce transpiration, consequently minimizing the loss of water and the depletion of carbon reserves, which are even more drastic in climacteric fruits due to ripening [52,58,59].

When fruits detach from the parent plant, respiration becomes their primary physiological process. Therefore, controlling this phenomenon is crucial for maintaining the quality and extending the shelf life of highly perishable fruits [60,61]. The rate at which fruit loses water is related to the pressure gradient between the plant tissues and the surrounding atmosphere [62]. Acerola is a species well-adjusted to temperatures around 5 °C and relative humidities above 85%, criteria fulfilled by the adopted storage conditions, which led to efficient preservation of the water content of the fruits over time.

Studies have shown that ozone may induce different responses regarding fresh mass loss. In papayas [63], strawberries [64], and pitayas [65], ozone reduced the fresh mass loss during storage compared with the control groups. On the other hand, in grapes [66] and blueberries [46], the fresh mass loss of the ozone-treated fruits increased. This variable was not influenced in lychees immersed in ozonated water (0.08 mg L^−1^ for 12 min) [67], similar to what was observed in this study.

Firmness is an important quality indicator for marketing fruits. Changes in this variable are typically linked to maturation, senescence, or existing contamination [60]. The preservation of this characteristic provides resistance during transportation and storage. It is also fundamental to design appropriate packaging that enhances protection against environmental factors, such as temperature and humidity, and mechanical damage resulting from compression, impact, vibration, and abrasion [60].

The loss of cell turgor, and consequently firmness, is inevitable during maturation and senescence. This process is characterized by the decomposition and depolymerization of molecules that confer rigidity to plant cells including pectin, hemicellulose, and cellulose [68,69]. The efficient maintenance of firmness in the presence of ozone is often associated with less breakdown of polysaccharides of the cell wall by solubilization. This is due to the inhibition of ethylene gas and enzymes such as α-arabinopyranosidase and β-galactopyranosidase [70]. The efficiency of ozone-based treatments in preserving firmness has also been verified in other climacteric fruits including mulberries (ozone gas at 0.00428 mg L^−1^) [71], kiwis (ozone gas at 0.15 mg L^−1^ during the day and 0.18 mg L^−1^ at night for 42 days) [72], and papayas (ozonated water at 0.8 mg L^−1^ for 40 to 160 min) [45].

The results of this study for soluble solids, pH, and total titratable acidity comply with Normative Ruling Number 37 of the Brazilian Ministry of Agriculture, Livestock, and Supply. It establishes the standards of identity and quality for ripe acerola pulp, which must have a minimum content of total soluble solids of 5.50%, pH of 2.80, and total titratable acidity of 0.80 citric acid % 100 g^−1^ [73].

Climacteric fruits like acerola accumulate a large amount of starch during maturation. This starch is later converted into sugars (glucose, sucrose, and fructose), thus augmenting the soluble solid values [74]. This process is triggered by increased respiratory rate and ethylene production, a hormone activating chlorophyllases and oxidases. These enzymes break down chlorophyll molecules and stimulate carotenogenesis, giving new colors to the fruit epidermis [60,74]. In the senescence phase, sugars are consumed for energy production in respiration, considerably depleting the soluble solid content [74].

In this study, no significant changes were observed in the total soluble solid content during storage, and all treatments exhibited a similar trend and variation range for this variable. Although significant differences and oscillations in the total soluble solid content were found among the treatments, the legal quality standard of the acerolas was not influenced by the presence of ozone microbubbles or ozone-free microbubbles.

Other studies have found different behaviors for the total soluble solids in ozonated climacteric fruits. Throughout the storage of bananas [52] and papayas [63], the total soluble solid content of the fruits increased up to the sixth and eighth days, respectively. After that, the values decreased due to overripening or senescence of the fruits. Nonetheless, ozone treatments were efficient in preserving this characteristic.

The total titratable acidity and pH followed a typical pattern of many climacteric fruits. After harvesting, the pH values tend to decrease initially but then increase as the fruit ripens or enters the senescence process. After maturation, high pH values and low citric acid content indicate advanced senescence and insalubrity stages. Organic acids (citric, malic, and tartaric acids) act as preserving agents by reducing the pH. These acids build up as the fruit grows and are then utilized as substrates for respiration, leading to a pH rise during ripening [75]. The increase in pH (from acidic to basic) and the decrease in total titratable acidity are related to the oxidation of pyruvic acid. This acid is a product of starch being broken down into reducing sugars, a process that occurs in the Krebs cycle during respiration [60].

A similar trend was observed in a study that exposed kiwis to ozone gas [76]. As the fruits ripened, the pH increased, while the total titratable acidity decreased, and ozone did not negatively affect these variables. This same effect occurred in mulberries, where the total titratable acidity naturally declined during ripening. However, the ozonated fruits presented higher levels of citric acid and a lower decomposition rate than the control group [71].

The reduction in vitamin C content seen during storage is part of a natural process during acerola ripening. The more advanced the maturation stage, the lower these levels. This decrease results from the oxidation of ascorbic acid into dehydroascorbic acid, a reaction catalyzed by the enzyme ascorbate oxidase found within the cell’s apoplasts [77,78].

Acerolas are naturally rich in vitamin C, thereby their nutritional relevance. The optimal values range from 1000 to 2000 ascorbic acid mg 100 g^−1^ in ripe acerolas, but they can exceed 3000 ascorbic acid mg 100 g^−1^ in green or partially ripe fruits [16] (pp. 27–47) [8]. In this research, the 20-min ozone microbubble treatment was the only one capable of preserving the vitamin C of the fruits within the optimal range up to the ninth day of storage (1121.00 ascorbic acid mg 100 g^−1^).

In Brazil, ripe acerolas have to contain a minimum of 800.00 ascorbic acid mg 100 g^−1^ [73], but the content must exceed 1200.00 ascorbic acid mg 100 g^−1^ to meet the export criteria [79]. In this case, the 20-min ozone treatment proved more efficient in meeting the international standards. On storage day 6, the vitamin C content in this treatment was 1242.00 ascorbic acid mg 100 g^−1^, which was within the international requirements.

This research demonstrated that exposing acerolas to ozone for too long tended to significantly reduce the vitamin C content during storage compared with the control. It also showed that prolonged contact with ozone caused the most harm to the fruit skin. However, other studies have reported the positive effects of ozone on vitamin C. The use of ozone gas in mulberries [80] and raspberries [77] as well as ozonated water in strawberries [50,59,64] resulted in high levels of vitamin C throughout storage. In these studies, the vitamin C levels also decreased over time, but they were considerably higher in the ozone-treated fruits than in the untreated ones. In turn, oranges and tangerines under continuous exposure to ozone gas retained their vitamin C content practically unchanged throughout storage [81].

#### 4.2.2. Pulp Color, Total Phenolic Compounds, and Total Antioxidant Activity (ABTS and DPPH)

Color alterations are first caused by the natural ripening process, as the distance from the initial coloration becomes greater over storage. The breakdown of anthocyanins may intensify these changes. In acerola pulp, the orange or yellow coloration from carotenoids is overlapped by the red anthocyanins in the skin, notably malvidin-3,5-diglucoside. The color hue decreases as the fruits mature, indicating a change from green/yellow to red, influenced by anthocyanin production [82]. On the other hand, the increased hue demonstrates a more significant yellowing. The closer the processed pulp is to orange or yellow, the less opaque the coloring, and the higher the hue value [83]. This may explain the increased hue (h*) and chroma (C*) in acerola pulp during storage. Color changes due to skin damage and pigment degradation were also verified on the surface of papayas [84], lychees [85], and strawberries [57] and in the processed peach pulp [86] exposed to ozone gas.

The oscillation in the phenolic compound content and antioxidant capacity during storage is related to the ability to protect against reactive oxygen species produced after the fruit treatment. This antioxidant defense is triggered by enzymatic reactions, with the increased activity of antioxidant enzymes such as superoxide dismutase, catalase, and ascorbate peroxidase [84,87]. Reactive oxygen species also prompt phenylalanine ammonia-lyase activity, the main enzyme involved in phenolic biosynthesis [63]. Its augmented activity results in the accumulation of phenolic compounds and enhances antioxidant capacity by deaminating L-phenylalanine into trans-cinnamic acid [88]. Compounds from the antioxidant group, such as phenolic acids, flavonoids, and tannins, preserve cellular redox homeostasis by interacting with reactive oxygen species and oxidation products [88].

The metabolic response of fruits to oxidative stress caused by ozone depends on factors such as concentration, exposure time, application method, nature of the product, and type of phenolic compound as well as its bioavailability [89,90]. The phenolic compound content may increase as a response to oxidative stress or decrease to avoid damage to other compounds such as DNA, lipids, and proteins [91]. This study revealed that exposing acerolas to ozonated water for 20 min might have activated their defense mechanisms against oxidative stress. However, longer exposures (40 and 60 min) may have diminished the antioxidant content and constrained the fruit’s defense capacity.

The antioxidant defense of fruits against ozone may also manifest through non-enzymatic reactions, increasing the content of antioxidant substances such as ascorbic acid and glutathione peroxidase [92,93]. In acerolas, vitamin C is the most abundant antioxidant, and it acts by eliminating, delaying, or inhibiting reactive oxygen species [94]. In oxidative stress by ozone, especially at high concentrations or prolonged exposure, the enzyme ascorbate oxidase boosts ascorbic acid oxidation into dehydroascorbic acid, which can then be irreversibly oxidized into diketogulonic acid. As a result, vitamin C decreases in the fruits, and its bio-efficacy is compromised [95,96]. This reaction likely explains the sharp decline in the ascorbic acid content over storage observed for the longer exposures to ozone microbubbles (T3 and T4/40 and 60 min), as opposed to the more gradual reductions in phenolic compounds and antioxidant activity.

This behavior may also explain the loss of the antioxidant activity of carotenoids. Lutein, violaxanthin, α-carotene, β-carotene, and β-cryptoxanthin are the main carotenoids in acerola pulp, with β-carotene making up more than 90% of the total content [97,98]. Carotenoids also protect against oxidative stress caused by reactive oxygen species, nitrogen, and lipid peroxides, preventing the formation of singlet oxygen and free radicals by ozonation [99]. High ozone concentrations or prolonged exposures can decrease these pigments through oxidative cleavage, increasing the activity of the enzyme 9-cis-epoxycarotene and the production of abscisic acid, thereby accelerating senescence [63].

Ozone can enhance the antioxidant defense mechanism at adequate concentrations and exposure durations, providing a more efficient rotting control [84]. This is because the accumulation of total phenolic compounds leads to an increase in phytoalexins, phenolic compounds with antimicrobial activity that build up locally in response to abiotic stresses [63]. This and other factors, such as the greater preservation of vitamin C, pH, citric acid content, phenolic compounds, and total antioxidant activity, may explain why the 20-min ozone treatment was the most effective for microbial decontamination.

The influence of ozone gas exposure on the total phenolic compounds and antioxidant activity was also investigated in processed melons [100]. When exposing the fruits for 30 and 60 min, ozone gas caused a more significant accumulation of phenolic compounds than the control group. Additionally, expanding the treatment duration from 30 to 60 min impacted the loss of total antioxidant activity as a result of the degradation of color pigments and vitamin C. A similar trend was observed in raspberries exposed to ozone gas for 60 and 120 min, with greater efficiency in microbiological decontamination in the shorter exposure time [77]. In mulberries, ozone gas (0.00428 mg L^−1^) extended the shelf life of the fruits, preserved their qualitative attributes, and conferred better resistance to pathogens by inducing the production of phytoalexins, resveratrol, and pterostilbene [71].

#### 4.2.3. External Appearance of Acerolas During Storage

Initially, damage to the fruit skin is probably linked to the oxidation of the cuticular wax. The outer protective layer of the acerolas is delicate, with only 10 to 30 total wax µg cm^−2^ [101]. Removing this protective layer may also have favored the degradation of color pigments in the skin. Previous studies have demonstrated the importance of cuticular wax in maintaining the postharvest quality of fruits [102,103,104] and its influence on fruit preservation when associated with ozone-based treatments [105]. However, the occurrence of external damage also depends on the ozone concentration, exposure time, and fruit skin thickness.

Applying ozone via microbubbles might have contributed to the fruit skin damage as it enhances the oxidative potential of ozone [106]. Low temperatures intensify these effects, in addition to helping extend the ozone half-life [106,107]. In this study, the temperature of 7 ± 1 °C was adopted during the immersion of the fruits in ozonated water. In this case, the use of microbubbles associated with the temperature of the treatment may have increased the oxidation capacity of ozone. Fruits such as apples [33], strawberries, cherries, and apricots [34] showed a different behavior when immersed in ozonated water with a microbubble system. In these studies, no changes were observed in the epidermis of the fruits after ozonation.

Although the sanitization of fruits with ozonated water in a microbubble system is a promising technology, some aspects must be considered in commercial-scale treatment systems [108]. First, the presence of by-products of oxidation caused by ozone in the treatment water must be monitored. Studies indicate that water with significant concentrations of bromide can lead to the formation of bromate, which is carcinogenic [109]. In this sense, the presence of this compound in the water to be ozonated must be observed. The construction of ozonated water generation systems in microbubble systems must also prioritize the occupational safety of employees, as ozone can also be toxic and harmful to human health [110]. The development of large-scale treatment systems must consider these harmful effects on health, reinforcing the need to follow safety protocols. Furthermore, the efficiency of ozone incorporation into water is directly impacted by water temperature, pH, and the presence of organic matter [111]. These are aspects that should also be considered when carrying out future experiments and applications of this technology as well as economic feasibility studies for each type of product.

The present research demonstrated that utilizing ozonated water with a microbubble system might be a promising alternative for treating acerolas before processing. However, further adjustments are necessary to employ this method for preserving fruits intended for fresh consumption. Additionally, as visual quality is an important aspect when marketing fresh acerolas, the duration of the ozonation process should be carefully considered due to its potential to cause undesirable changes in fruit color.

## 5. Conclusions

Treating acerolas with ozonated water using a microbubble system for 20 min proved to be the most effective for bacterial and fungal decontamination as well as for the preservation of vitamin C, pH, total titratable acidity, total phenolic compounds, and total antioxidant activity throughout storage. This technique also successfully maintained the firmness of the fruits, regardless of the exposure duration. On the other hand, the longer the immersion into ozonated water, the greater the damage to the skin of the fruits.

## Figures and Tables

**Table 1:** Mean values for the aerobic mesophile count and filamentous fungi and yeast count in acerolas treated with ozone microbubbles or ozone-free microbubbles; treatments were evaluated throughout storage.

	Aerobic Mesophiles (log CFU g^−1^)			
Treatment	0	3	6	9
T1	4.42	4.03	Uncountable	Uncountable
T2	3.65	<2.0 log (CFU g^−1^)	4.79	3.40
T3	3.70	4.92	5.52	4.84
T4	3.43	4.53	4.25	4.23
T5	3.76	5.54	Uncountable	Uncountable
T6	3.71	5.19	Uncountable	Uncountable
T7	5.09	5.10	Uncountable	Uncountable
**Treatment**	**Filamentous Fungi and Yeasts (log CFU g^−1^)**			
T1	<2.0 log (CFU g^−1^)	5.04	Uncountable	Uncountable
T2	<2.0 log (CFU g^−1^)	<2.0 log (CFU g^−1^)	5.76	6.77
T3	<2.0 log (CFU g^−1^)	5.32	6.04	6.02
T4	4.32	4.45	4.63	4.73
T5	<2.0 log (CFU g^−1^)	5.36	Uncountable	Uncountable
T6	<2.0 log (CFU g^−1^)	6.23	Uncountable	Uncountable
T7	4.41	5.50	Uncountable	Uncountable

Treatments: T1—control (untreated fruits); T2—ozone microbubble/20 min; T3—ozone microbubble/40 min; T4—ozone microbubble/60 min; T5—ozone-free microbubble/20 min; T6—ozone-free microbubble/40 min; T7—ozone-free microbubble/60 min. Unc.: uncountable, over the limit. Values exceeding the count range, with a maximum limit of 250 colonies for aerobic mesophiles, and 150 colonies for filamentous fungi and yeasts.

**Table 2:** Mean values of fresh mass loss, firmness, total soluble solid content, potential of hydrogen, total titratable acidity and vitamin C.

	Fresh Mass Loss (%)			
	Storage (Days)			
Treatment	0	3	6	9
T1	-	1.25 a	2.41 a	3.91 a
T2	-	1.42 a	2.67 a	3.95 a
T3	-	1.37 a	2.49 a	3.96 a
T4	-	1.12 a	2.78 a	4.19 a
T5	-	1.38 a	2.54 a	4.15 a
T6	-	1.55 a	2.54 a	4.01 a
T7	-	1.21 a	2.41 a	3.97 a
**Treatment**	**Firmness (N)**			
T1	0.51 a	0.52 ab	0.27 c	0.11 c
T2	0.46 a	0.46 abc	0.46 ab*	0.27 b*
T3	0.48 a	0.48 abc	0.35 bc	0.25 b*
T4	0.55 a	0.54 a	0.52 a*	0.37 a*
T5	0.51 a	0.29 d*	0.29 c	0.11 c
T6	0.52 a	0.38 bcd*	0.30 c	0.13 c
T7	0.41 a	0.34 cd*	0.35 bc	0.14 c
**Treatment**	**Total soluble solid content (%)**			
T1	10.60 a	10.83 ab	9.57 c	9.30 c
T2	9.70 b*	10.47 b	9.77 bc	9.33 c
T3	8.73 d*	10.67 ab	9.50 c	10.10 b*
T4	9.20 c*	10.80 ab	10.10 b*	10.17 ab*
T5	10.00 b*	11.27 a	10.57 a*	10.67 a*
T6	9.83 b*	10.63 ab	10.07 b*	10.63 ab*
T7	9.70 b*	10.30 b	9.10 d*	9.10 c
**Treatment**	**Potential of Hydrogen (pH)**			
T1	2.90 b	2.70 c	3.10 a	3.10 a
T2	2.57 d*	2.70 c	2.90 b*	2.90 c*
T3	2.77 c*	2.80 b	2.87 b*	3.00 b
T4	2.80 c*	2.60 d	3.10 a	3.00 b
T5	2.90 b	2.90 a*	3.10 a	3.00 b
T6	2.90 b	2.90 a*	3.07 a	3.00 b
T7	3.00 a*	2.90 a*	3.10 a	3.00 b
**Treatment**	**Total titratable acidity (citric acid)**			
T1	2.53 ab	2.93 a	2.11 a	1.85 a
T2	2.55 a	2.18 b*	2.07 a	1.81 a
T3	2.32 c*	1.95 e*	1.78 bc*	1.58 c*
T4	2.53 ab	2.09 c*	1.83 b*	1.69 b*
T5	2.44 b	1.54 f*	1.87 b*	1.59 c*
T6	2.22 c*	2.03 d*	1.69 c*	1.57 c*
T7	2.03 d*	2.07 cd*	1.88 b*	1.87 a
**Treatment**	**Vitamin C (ascorbic acid)**			
T1	1673.14 a	1585.08 a	1135.97 b	951.05 b
T2	1617.37 ab	1435.38 b*	1241.65 a*	1121.30 a*
T3	1661.40 a	1100.75 d*	1012.69 c*	789.60 c*
T4	1602.69 ab	1138.91 c*	883.54 d*	786.67 c*
T5	1361.99 bc*	1091.94 d*	821.89 e*	636.97 d*
T6	1479.41 abc	1015.63 e*	774.93 e*	545.97 e*
T7	1309.16 c*	1162.39 c*	983.34 c*	968.66 b

Means followed by the same lowercase letter in the column did not differ according to Tukey’s test at a 5% probability level. Means followed by an asterisk in the column differed from the control group according to Dunnett’s test at a 5% probability level. Treatments: T1—control (untreated fruits); T2—ozone microbubble/20 min; T3—ozone microbubble/40 min; T4—ozone microbubble/60 min; T5—ozone-free microbubble/20 min; T6—ozone-free microbubble/40 min; T7—ozone-free microbubble/60 min.

**Table 3:** Adjusted regression equations and coefficients of determination (R^2^/r^2^) for the fresh mass loss, firmness, total soluble solids, pH, total titratable acidity, and vitamin C of acerolas as a function of storage time (ST) and exposure time (ET).

Variable	Treatment	Adjusted Equations	R^2^/r^2^
Fresh mass loss (%)	Control (untreated fruits) (T1)	${\hat{y}}_{i}=$ −0.144442 + 0.4447 ** ST	0.9422
	Ozone microbubble (T2, T3, and T4)	${\hat{y}}_{i}=$ −0.07661 + 0.45513 ** ST	0.9884
	Ozone-free microbubble (T5, T6, and T7)	${\hat{y}}_{i}=$ −0.02336 + 0.44007 ** ST	0.9836
**Variable**	**Treatment**	**Adjusted equations**	**R^2^/r^2^**
Firmness (N)	Control (untreated fruits) (T1)	${\hat{y}}_{i}=$ 0.567 − 0.04766 ** ST	0.8827
	Ozone microbubble (T2, T3, and T4)	${\hat{y}}_{i}=$ 0.609611 + 0.01487 ^ns^ ST − 0.0040432 ** ST^2^ − 0.00977 * ET + 0.0001468 * ET^2^	0.9312
	Ozone-free microbubble (T5, T6, and T7)	${\hat{y}}_{i}=$ 0.4768 − 0.03622 ** ST	0.8707
**Variable**	**Treatment**	**Adjusted equations**	**R^2^/r^2^**
Total soluble solids content (%)	Control (untreated fruits) (T1)	${\hat{y}}_{i}=$ 10.85 − 0.17222 * ST	0.7806
	Ozone microbubble (T2, T3, and T4)	${\hat{y}}_{i}=$ 9.87	-
	Ozone-free microbubble (T5, T6, and T7)	${\hat{y}}_{i}=$ 10.15	-
**Variable**	**Treatment**	**Adjusted equations**	**R^2^/r^2^**
Potential of Hydrogen (pH)	Control (untreated fruits) (T1)	${\hat{y}}_{i}=$ 2.95	-
	Ozone microbubble (T2, T3, and T4)	${\hat{y}}_{i}=$ 2.68 + 0.03407 ** ST	0.5467
	Ozone-free microbubble (T5, T6, and T7)	${\hat{y}}_{i}=$ 2.98	-
**Variable**	**Treatment**	**Adjusted equations**	**R^2^/r^2^**
Total titratable acidity (citric acid)	Control (untreated fruits) (T1)	${\hat{y}}_{i}=$ 2.35	-
	Ozone microbubble (T2, T3, and T4)	${\hat{y}}_{i}=$ 2.41 − 0.08377 ** ST	0.8503
	Ozone-free microbubble (T5, T6, and T7)	${\hat{y}}_{i}=$ 2.1587 − 0.05754 ** ST	0.5380
**Variable**	**Treatment**	**Adjusted equations**	**R^2^/r^2^**
Vitamin C (ascorbic acid)	Control (untreated fruits) (T1)	${\hat{y}}_{i}=$ 1728.62 − 87.1794 * ST	0.9398
	Ozone microbubble (T2, T3, and T4)	${\hat{y}}_{i}=$ 1804.74 − 62.742 ** ST − 7.8764 ** ET	0.8753
	Ozone-free microbubble (T5, T6, and T7)	${\hat{y}}_{i}=$ 1347.02 − 74.296 ** ST	0.8178

** Significant at a 1% probability level according to the Student’s *t*-test. * Significant at a 5% probability level according to the Student’s *t*-test. ^ns^ Non-significant. Treatments: T1—control (untreated fruits); T2—ozone microbubble/20 min; T3—ozone microbubble/40 min; T4—ozone microbubble/60 min; T5—ozone-free microbubble/20 min; T6—ozone-free microbubble/40 min; T7—ozone-free microbubble/60 min.

**Table 4:** Values of color difference, color hue color saturation, total phenolic compounds and total antioxidant activity (ABTS and DPPH).

	Color Difference (Dif*)			
	Storage (Days)			
Treatment	0	3	6	9
T1	0.83 d	2.27 b	4.83 b	10.17 c
T2	4.19 a*	2.21 b	2.96 cde*	12.41 b*
T3	3.23 b*	2.72 ab	2.14 e*	14.11 a*
T4	4.06 ab*	3.57 ab	4.00 bc	14.16 a*
T5	1.85 c*	2.42 b	2.36 de*	10.92 c
T6	0.94 d	3.51 ab	3.29 cd*	11.01 c
T7	1.12 cd	4.07 a*	6.60 a*	14.23 a*
**Treatment**	Color hue (h*)			
T1	22.43 e	26.06 c	26.96 e	33.04 de
T2	24.92 bc*	28.21 b*	28.72 cd*	40.44 b*
T3	27.75 a*	28.24 b*	30.04 b*	42.36 a*
T4	23.95 d*	30.41 a*	32.22 a*	40.25 b*
T5	24.55 bcd*	29.73 ab*	28.39 d*	34.49 c*
T6	25.26 b*	28.40 b*	28.98 cd*	33.74 cd
T7	24.28 cd*	29.03 ab*	29.58 bc*	32.48 e
**Treatment**	Color saturation (C*)			
T1	11.90 cd	13.55 d	15.61 a	17.56 c
T2	15.09 a*	15.71 b*	15.56 a	22.57 a*
T3	13.80 b*	15.70 b*	12.47 c*	22.96 a*
T4	14.83 a*	17.09 a*	15.93 a	23.17 a*
T5	13.38 b*	13.14 d	13.50 bc*	19.34 b*
T6	12.11 c	14.78 bc*	13.51 b*	17.98 c
T7	11.16 d	14.15 cd	15.86 a	20.65 b*
**Treatment**	**Total phenolic compounds (gallic acid)**			
T1	2392.98 ab	1727.81 a	1481.28 ab	1412.35 ab
T2	2672.61 a	1686.00 a	1503.89 a	1487.73 a
T3	2154.00 b	1363.68 c*	1268.81 c*	1214.07 bc
T4	2537.98 ab	1547.42 b*	1415.32 b	1292.37 ab
T5	1617.19 c*	1525.00 b*	1475.75 ab	1041.18 c*
T6	1604.04 c*	1520.04 b*	1451.89 ab	1365.30 ab
T7	1517.11 c*	1492.39 b*	1481.05 ab	1415.84 ab
**Treatment**	**Total antioxidant activity ABTS (Trolox)**			
T1	9814.48 a	7719.56 abc	7433.29 ab	6943.30 ab
T2	8656.72 b*	8536.92 a	8199.30 a	6665.47 b
T3	7206.77 c*	7016.03 bc	4136.04 d*	4062.47 e*
T4	9204.84 ab*	7672.56 abc	7150.03 abc	6138.27 c*
T5	9181.46 ab*	8074.13 ab	7067.67 abc	7045.56 a
T6	6954.81 c*	6855.22 c	6754.25 bc	6064.99 c*
T7	9323.78 a	7453.67 abc	6139.41 c*	4645.77 d*
**Treatment**	**Total antioxidant activity DPPH (Trolox)**			
T1	429.74 d	245.06 e	217.86 d	170.68 c
T2	893.39 a*	575.17 a*	429.33 a*	392.19 a*
T3	782.04 b*	269.34 de	266.45 c*	144.69 cd*
T4	669.19 c*	493.10 b*	365.96 b*	108.19 e*
T5	635.94 c*	504.06 b*	372.18 b*	153.69 c
T6	773.11 b*	319.82 d*	218.48 d	117.14 de*
T7	870.78 a*	424.28 c*	356.45 b*	236.04 b*

Means followed by the same lowercase letter in the column do not differ according to Tukey’s test at a 5% probability level. Means followed by an asterisk in the column differ from the control group according to Dunnett’s test at a 5% probability level. Treatments: T1—control (untreated fruits); T2—ozone microbubble/20 min; T3—ozone microbubble/40 min; T4—ozone microbubble/60 min; T5—ozone-free microbubble/20 min; T6—ozone-free microbubble/40 min; T7—ozone-free microbubble/60 min.

**Table 5:** Adjusted regression equations and coefficients of determination (R^2^/r^2^) for the color variables, total phenolic compounds, and total antioxidant activity (ABTS and DPPH) as a function of storage time (ST) and exposure time (ET).

Variable	Treatment	Adjusted Equations	R^2^/r^2^	
Pulp color difference (Dif*)	Control (untreated fruits) (T1)	${\hat{y}}_{i}=$ −0.05879 + 1.01923 * ST	0.9223	
	Ozone microbubble (T2, T3, and T4)	${\hat{y}}_{i}=$ 4.2844 − 1.9016 ** ST + 0.320179 ** ST^2^	0.9283	
	Ozone-free microbubble (T5, T6, and T7)	${\hat{y}}_{i}=$ 0.4418 − 0.38436 ^ns^ ST + 0.16498 ** ST^2^ + 0.0006872 * ET	0.9096	
**Variable**	**Treatment**	**Adjusted equations**	**R^2^/r^2^**	
Pulp color hue (h*)	Control (untreated fruits) (T1)	${\hat{y}}_{i}=$ 22.2174 + 1.09059 * ST	0.9203	
	Ozone microbubble (T2, T3, and T4)	${\hat{y}}_{i}=$ 26.107 − 0.2245 ^ns^ ST + 0.20199 ** ST^2^	0.9068	
	Ozone-free microbubble (T5, T6, and T7)	${\hat{y}}_{i}=$ 25.094 + 0.8848 ** ST	0.8644	
**Variable**	**Treatment**	**Adjusted equations**	**R^2^/r^2^**	
Pulp color saturation (C*)	Control (untreated fruits) (T1)	${\hat{y}}_{i}=$ 11.7931 + 0.63554 ** ST	0.9979	
	Ozone microbubble (T2, T3, and T4)	${\hat{y}}_{i}=$ 15.2178 − 0.8811 ^ns^ ST + 0.184778 ** ST^2^	0.7702	
	Ozone-free microbubble (T5, T6, and T7)	${\hat{y}}_{i}=$ 12.5312 − 0.0866 ^ns^ ST + 0.08958 * ST^2^	0.8201	
**Variable**	**Treatment**	**Adjusted equations**	**R^2^/r^2^**	
Total phenolic compounds (gallic acid)	Control (untreated fruits) (T1)	${\hat{y}}_{i}=$ 2231.87 − 106.281 * ST	0.8470	
	Ozone microbubble (T2, T3, and T4)	${\hat{y}}_{i}=$ 2419.15 − 331.364 ** ST + 23.83 ** ST^2^	0.8752	
	Ozone-free microbubble (T5, T6, and T7)	${\hat{y}}_{i}=$ 1458.89	-	
**Variable**	**Treatment**	**Adjusted equations**	**R^2^/r^2^**	
Total antioxidant activity ABTS	Control (untreated fruits) (T1)	${\hat{y}}_{i}=$ 9312.63 − 296.66 * ST	0.8239	
	Ozone microbubble (T2, T3, and T4)	${\hat{y}}_{i}=$ 16186.6 − 314.961 ** ST − 446.367 ** ET + 5.43172 ** ET^2^	0.8937	
	Ozone-free microbubble (T5, T6, and T7)	${\hat{y}}_{i}=$ 8406.7 − 283.699 ** ST	0.5853	
**Variable**	**Treatment**	**Adjusted equations**	**R^2^/r^2^**	
Total antioxidant activity DPPH	Control (untreated fruits) (T1)	${\hat{y}}_{i}=$ 386.493 − 26.8124 * ST	0.8369	
	Ozone microbubble (T2, T3, and T4)	${\hat{y}}_{i}=$ 881.22 − 59.7168 ** ST + 4.0851 * ET	0.8108	
	Ozone-free microbubble (T5, T6, and T7)	${\hat{y}}_{i}=$ 696.164 − 62.4442 * ST	0.8292	

** Significant at a 1% probability level according to Student’s *t*-test. * Significant at a 5% probability level according to Student’s *t*-test. ^ns^ Non-significant. Treatments: T1—control (untreated fruits); T2—ozone microbubble/20 min; T3—ozone microbubble/40 min; T4—ozone microbubble/60 min; T5—ozone-free microbubble/20 min; T6—ozone-free microbubble/40 min; T7—ozone-free microbubble/60 min.

## Data Availability

The original contributions presented in the study are included in the article, further inquiries can be directed to the corresponding author.

## Supplementary Materials

The following supporting information can be downloaded at: https://www.mdpi.com/article/10.3390/foods14101814/s1, Tables S1–S4: Titles. Table S1. Mean values for aerobic mesophiles count and filamentous fungi and yeasts count; Results obtained in the preliminary assay of acerola fruits, immediately after treatment; Table S2. Mean values of firmness and vitamin C. Results obtained in the preliminary assay of acerola fruits, immediately after treatment; Table S3. Mean values of soluble solids content, potential of hydrogen and total titratable acidity. Results obtained in the preliminary assay of acerola fruits, immediately after treatment; Table S4. Mean values of color difference, color hue and color saturation in the acerola pulp. Results obtained in the preliminary assay of acerola fruits, immediately after treatment.
