# Supplementary material for: Efficiency of Ozonated Water Treatment with a Microbubble System for Sanitization and Preservation of Postharvest Quality of Acerolas
Source: Foods. 2025 May 20;14(10):1814. doi: 10.3390/foods14101814 (PMC12111660; doi:10.3390/foods14101814)
Supplement: Supplementary file 1 [file foods-14-01814-s001.zip › foods-3618870-supplementary.pdf]

Supplementary Material

Article: Efficiency of ozonated water treatment with a microbubble system for sanitization and preservation of postharvest quality of acerolas

Table S1. Mean values for aerobic mesophiles count and filamentous fungi and yeasts count. Results obtained in the preliminary assay of acerola fruits, immediately after treatment.

Treatments	Aerobic mesophiles ($\log CFU\ g^{-1}$)	Filamentous fungi and yeasts ($\log CFU\ g^{-1}$)
Control (untreated fruits)	5.17	4.26 ± 0.00
Ozone microbubble/5 min	5.02	< 2.0 log CFU g^{-1}
Ozone microbubble/10 min	5.29	< 2.0 log CFU g^{-1}
Ozone microbubble/20 min	4.53	< 2.0 log CFU g^{-1}
Ozone microbubble/30 min	4.39	< 2.0 log CFU g^{-1}
Ozone-free microbubble/5 min	5.24	4.29 ± 0.10
Ozone-free microbubble /10 min	5.19	4.19 ± 0.01
Ozone-free microbubble /20 min	4.99	4.19 ± 0.01
Ozone-free microbubble /30 min	5.11	4.59 ± 0.25

Means followed by the same lowercase letter in the column do not differ according to Tukey's test at a 5% probability level. Means followed by an asterisk in the column differ from the control group according to Dunnett's test at a 5% probability level.

Table S2. Mean values of firmness and vitamin C. Results obtained in the preliminary assay of acerola fruits, immediately after treatment.

Treatments	Firmness (N)	Vitamin C (mg 100 g^{-1} of pulp)
Control (untreated fruits)	0.20 b	1059.66 ab
Ozone microbubble/5 min	0.27 ab	1083.14 a
Ozone microbubble/10 min	0.24 ab	1042.04 abc
Ozone microbubble/20 min	0.28 ab	995.08 c*
Ozone microbubble/30 min	0.26 ab	1024.43 bc
Ozone-free microbubble/5 min	0.25 ab	1089.01 a
Ozone-free microbubble /10 min	0.36 a*	1089.01 a
Ozone-free microbubble /20 min	0.26 ab	1024.43 bc
Ozone-free microbubble /30 min	0.30 ab	1053.78 ab

Means followed by the same lowercase letter in the column do not differ according to Tukey's test at a 5% probability level. Means followed by an asterisk in the column differ from the control group according to Dunnett's test at a 5% probability level.

Table S3. Mean values of soluble solids content, potential of hydrogen and total titratable acidity. Results obtained in the preliminary assay of acerola fruits, immediately after treatment.

Treatments	Soluble solids (%)	Potential of Hydrogen (pH)	Titratable acidity (%)
Control (untreated fruits)	7.20 ab	3.27 ab	1.41 a
Ozone microbubble/5 min	7.27 a	3.20 b	1.37 a
Ozone microbubble/10 min	7.03 bc*	3.20 b	1.41 a
Ozone microbubble/20 min	7.13 abc	3.20 b	1.36 a
Ozone microbubble/30 min	7.13 abc	3.30 a	1.35 a
Ozone-free microbubble/5 min	7.07 bc	3.30 a	1.43 a
Ozone-free microbubble /10 min	6.97 c*	3.23 ab	1.42 a
Ozone-free microbubble /20 min	6.97 c*	3.20 b	1.44 a
Ozone-free microbubble /30 min	7.10 abc	3.23 ab	1.43 a

Means followed by the same lowercase letter in the column do not differ according to Tukey's test at a 5% probability level. Means followed by an asterisk in the column differ from the control group according to Dunnett's test at a 5% probability level.

Table S4. Mean values of color difference, color hue and color saturation in the acerola pulp. Results obtained in the preliminary assay of acerola fruits, immediately after treatment.

Treatments	Color difference (Dif*)	Color hue (h*)	Color saturation (C*)
Control (untreated fruits)	1.49 ab	45.74 d	18.99 ± a
Ozone microbubble/5 min	1.72 ab	45.64 de	17.75 ± b*
Ozone microbubble/10 min	1.75 ab	44.69 ± e*	17.74 ± b*
Ozone microbubble/20 min	1.37 b	46.78 ± bc*	17.85 ± b*
Ozone microbubble/30 min	2.23 ab	46.92 ± bc*	17.79 ± b*
Ozone-free microbubble/5 min	2.18 ab	46.98 ± bc*	17.94 ± ab*
Ozone-free microbubble /10 min	2.64 a*	48.76 ± a*	18.27 ± ab
Ozone-free microbubble /20 min	2.37 ab	47.73 ± b*	18.02 ± ab*
Ozone-free microbubble /30 min	2.23 ab	46.40 ± cd	17.84 ± b*

Means followed by the same lowercase letter in the column do not differ according to Tukey's test at a 5% probability level. Means followed by an asterisk in the column differ from the control group according to Dunnett's test at a 5% probability level.
